# Clinical Research in Vulnerable Populations: Variability and Focus of Institutional Review Boards’ Responses

**DOI:** 10.1371/journal.pone.0135997

**Published:** 2015-08-14

**Authors:** Bärbel Kästner, Simone Behre, Nadine Lutz, Friederike Bürger, Steffen Luntz, Katrin Hinderhofer, Martin Bendszus, Georg F. Hoffmann, Markus Ries

**Affiliations:** 1 Coordination Center for Clinical Trials (KKS), University Hospital Heidelberg, Heidelberg, Germany; 2 Pediatric Neurology and Metabolic Medicine, Center for Rare Disease, Center for Pediatric and Adolescent Medicine, University Hospital Heidelberg, Heidelberg, Germany; 3 Institute for Human Genetics, University Hospital Heidelberg, Heidelberg, Germany; 4 Neuroradiology, Department of Neurology, University Hospital Heidelberg, Heidelberg, Germany; Texas Tech University Health Science Centers, UNITED STATES

## Abstract

**Background:**

Children and patients with cognitive deficits may find it difficult to understand the implication of research. In the European Union (EU), clinical studies outside the EU directives concerning medicinal products or medical devices, i.e., “miscellaneous clinical studies”, have no legally mandated timelines for institutional review boards’ (IRB) decisions.

**Goal:**

To evaluate the review process of IRBs for two different “miscellaneous” multicenter clinical research protocols involving vulnerable subjects (children and adult stroke patients).

**Methods:**

Descriptive and comparative statistics. Protocol 1 is a prospective, multicenter, cross-sectional screening study of a symptomatic pediatric population at risk for Fabry disease involving genetic testing (NCT02152189). Protocol 2 is a prospective, multicenter, randomized, controlled, open-label, blinded endpoint post-market study to evaluate the effectiveness of stent retrievers (NCT02135926). After having obtained positive initial IRB votes at the main study site, both protocols were subsequently submitted to the remaining IRBs.

**Results:**

Protocol 1 was submitted to 19 IRBs. No IRB objected to the study. Median time-to-final vote was 34 (IQR 10–65; range 0 to 130) days. Two IRBs accepted the coordinating center’s IRB votes without re-evaluation. Changes to the informed consent documents were asked by 7/19 IRBs, amendments to the protocol by 2. Protocol 2 was submitted to 16 IRBs. Fifteen decisions were made. No IRB objected to the study. Median time-to final vote was 59 (IQR 10 to 65; range 0 to 128) days, which was not statistically significantly different compared with protocol 1 (Wilcoxon test). Two IRBs accepted a previous IRB decision and did not conduct an independent review. Eight/16 IRBs required changes to the informed consent documents; two IRBs recommended an amendment of the protocol.

**Conclusion:**

Both clinical research protocols involving vulnerable populations were well accepted. IRB workflows and decision times varied substantially. Some IRBs accepted a previous IRB decision without the necessity of another reevaluation process. Requested changes were focused on the informed consent documents. A more standardized approach across jurisdictions is desirable.

## Introduction

Research can be associated with physical, psychological, social, legal, or economic risk, and cognitive, institutional, economic, and social factors can result in so-called vulnerabilities [1–4]. As such, certain subjects, e.g. children, prisoners, pregnant women, mentally disabled persons, economically or educationally disadvantaged persons may be vulnerable to coercion or undue influence. In addition, children or adults with cognitive deficits may find it difficult to understand the implications of research may therefore not be able to fully evaluate consent information [2]. Therefore, distinctive safeguards are included in subparts of the U.S. Code of Federal Regulations (45CFR46, subparts B–D) to protect the rights and welfare of these subjects [2,5]. Also the Declaration of Helsinki stresses the need of special protection for vulnerable groups and individuals: “Medical research with a vulnerable group is only justified if the research is responsive to the health needs or priorities of this group and the research cannot be carried out in a non-vulnerable group. In addition, this group should stand to benefit from the knowledge, practices or interventions that result from the research” [6]. A guidance document especially for clinical trials with the pediatric population was published by the European Commission [7]. Similar to the FDA who provides specific guidance for IRBs, Clinical Investigators, and Sponsors with regard to clinical trials as well as informed consent information sheets conducted in the U.S. [8], the European Commission publishes “The rules governing medicinal products in the European Union” that contain guidance documents applying to clinical trials [9].

In the European Union (EU), legally mandatory timelines for ethics review process are 60 days for studies involving medicinal products (EU Directive 2001/20/EC) and studies involving medical devices (Council Directive 93/42/EEC) [9,10]. For all other miscellaneous clinical studies, there are no legally mandated timelines. The precise knowledge of timelines and focus from institutional review boards to proposed miscellaneous clinical research protocols can be helpful in the planning and execution these trials. The purpose of this paper is to analyze a) the timelines of various IRBs’ assessments of research protocols involving vulnerable populations, b) the focus of IRB feedback to research protocols involving vulnerable populations c) contributing factors that can help explaining the heterogeneities in IRB responses to miscellaneous clinical studies outside of the two EU Directives.

## Material and Methods

We submitted two different multicenter studies involving vulnerable populations to various IRBs in Germany (N = 34) and Austria (N = 1). Study 1 was a prospective, cross-sectional screening study of a symptomatic pediatric population at risk for an inborn error of metabolism conducted at 29 clinical trial centers. In this study, patients with abdominal pain or pain in the extremities were screened for Fabry disease. Pediatric patients under 18 years were eligible. Whereas males were tested through an enzymatic assay, the study involved genetic testing in females. Target enrolment were N = 2000 patients. The trial was registered on clinicaltrials.gov under NCT02152189. The study population is considered vulnerable because it involves children.

Study 2 was a prospective, multicenter, randomized, controlled, open label, blinded endpoint post-market study to compare the safety and effectiveness of stent retrievers for thrombectomy compared to best medical treatment alone in acute ischemic stroke (AIS) patients not eligible for IV-tissue plasminogen activator treatment. The study was planned at 20 clinical trial centers. Males and females between 18 and 80 years were eligible. The study was registered as NCT02135926 on clinicaltrials.gov. The study population is considered vulnerable, because the participant’s ability to make fully informed decisions for himself may be compromised by the nature of the stroke which may interfere with cognition or consciousness.

Both protocols were initially submitted to the IRB at the coordinating sites. Then a local PI at the other trial sites was identified and after a positive vote at the coordinating sites’ IRBs the documents were subsequently submitted to the local IRBs. All study related materials were prepared by and submitted by the Coordination Center for Clinical Trials (KKS) of the University Hospital Heidelberg. In compliance with the local jurisdictions of the various study centers, the documents were either submitted to university IRBs or to state medical associations’ (“Landesärztekammer”) IRBs.

### Definitions and variables

The following variables were investigated: Time-to-final response, IRB type, number and nature of requested changes. The presentations of the IRBs’ websites were rated for their usability with regard to availability and presentation of necessary information to prepare IRB submission for both studies. Specifically, the website was expected to contain the following information: 1) which documents are necessary? 2) How many copies in which format (CD, paper) are required? 3) What is the pathway if a previous IRB vote is available? If all three items were available the website was rated as “good”, if only two criteria were fulfilled the rating was “medium”, and if only one or even none were fulfilled the rating was “bad”. The quality of the website was rated by the submitting individuals as they were considered the most appropriate raters with detailed knowledge for requirements of the submission.

### Statistical Analysis

We applied methods of descriptive and comparative statistics. Non-parametric tests (Wilcoxon, non-parametric ANOVA) were applied for not normally distributed data. A two-sided p of 0.05 or less was considered statistically significant. Continuous variables with non-parametric distribution were summarized with median, interquartile ranges (IQR) and overall ranges. All statistical analyses were performed using SAS Enterprise Guide version 9.1 (SAS, Cary, NC, USA).

## Results

### Variation in response time

**Fig 1:**
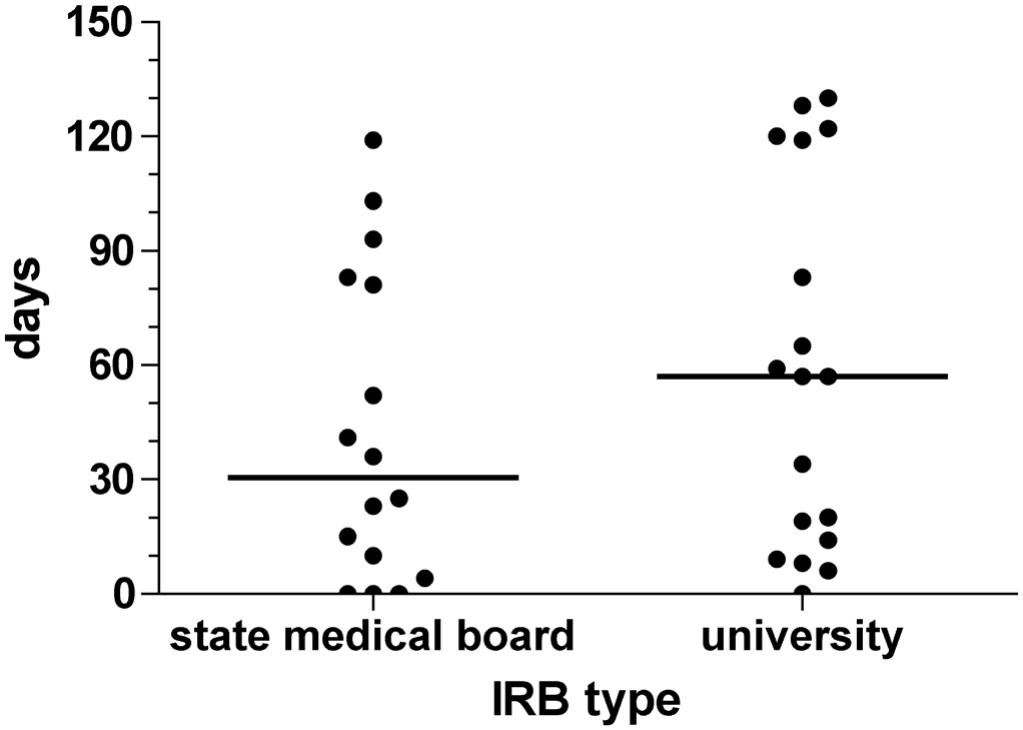
Time-to-final response [days] of two study submissions to 34 IRBs by IRB type (university or state medical board, p = NS). The overall median time-to-final response for the two studies was 38.5 (IQR 10 to 83; range 0 to 130) days.

Protocol 1 was submitted to 19 IRBs. No IRB objected to the study. Median time-to-final vote was 34 (IQR 10–65; range 0 to 130) days. Protocol 2 was submitted to 16 IRBs. Fifteen decisions were made. No IRB objected to the study, but the study was put on hold for scientific reasons before the 16^th^ IRB decision was made. Median time-to final vote was 59 (IQR 10 to 65; range 0 to 128) days, which was not statistically significantly different compared with protocol 1 (Wilcoxon test). The median time-to-final vote for university IRBs was 57 (IQR 14 to 119; range 0 to 130) days. State medical associations’ IRBs required a median time of 30.5 (IQR 7 to 82; range 0 to 119) days for a final vote. The differences between the two groups were not statistically significant (Fig 1).

### Focus and nature of requested changes

Protocol 1: Two IRBs accepted the coordinating center's IRB votes without re-evaluation. Eleven/19 IRBs did not request any changes. Changes to the informed consent documents were asked by 7/19 IRBs, amendments to the protocol by 2 IRBs.

Protocol 2: Two IRBs accepted a previous IRB decision and did not conduct an independent review. Eight/16 IRBs required changes to the informed consent documents; 2 IRBs recommended an amendment of the protocol.

**Fig 2:**
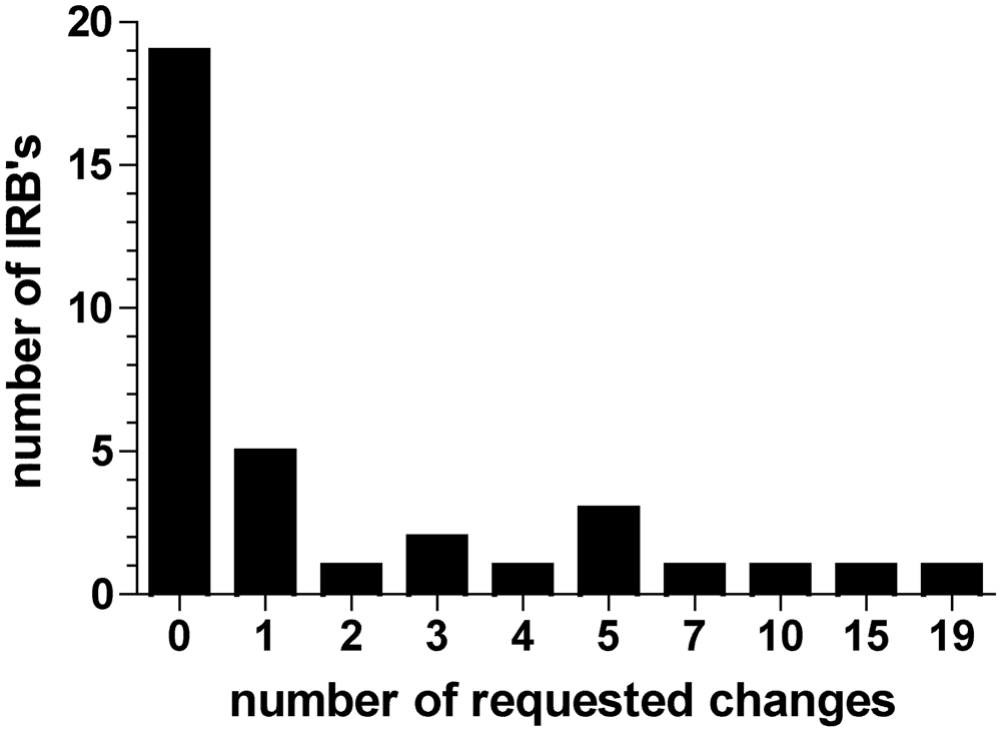
Total number of requested changes in the submission packages for two clinical studies submitted N = 35 IRBs.

The IRB at the coordinating sites drove the number of totally requested changes, i.e. 19 changes for study 1 and 15 changes for study 2. The subsequent submissions to the multicenter satellite sites required ≤ 10 total changes in the submission (Fig 2). Time-to-final response for the two coordinating sites’ IRBs was 57 days (study 1) and 122 days (study 2).

Overall, most changes (N = 65) were requested for the informed consent documents. Most requested changes were related to readability, syntax, usage, grammar and content information (Table 1).

**Table 1:** Nature of requested changes in the informed consent documents of two studies submitted to N = 35 IRBs.

	Study 1	Study 2	Both studies
Item	N (percentage)	N (percentage)	N (percentage)
Readability, syntax, usage, grammar	5 (23%)	18 (42%)	23 (35%)
Content information	7 (32%)	8 (19%)	15 (23%)
Data protection	4 (18%)	7 (16%)	11 (17%)
Obtaining consent	0 (0%)	6 (14%)	6 (9%)
Sample storage	5 (23%)	1 (2%)	6 (9%)
Risk about intervention	1 (4%)	1 (2%)	2 (3%)
Format of the document, layout, letter head	0 (0%)	2 (5%)	2 (3%)
Total	22 (100%)	43 (100%)	65 (100%)

The suggested changes to the protocol were either clarifications of wordings or more general scientific discussions that did not lead to a protocol amendment.

### Accessibly and transparency of IRB processes

**Fig 3:**
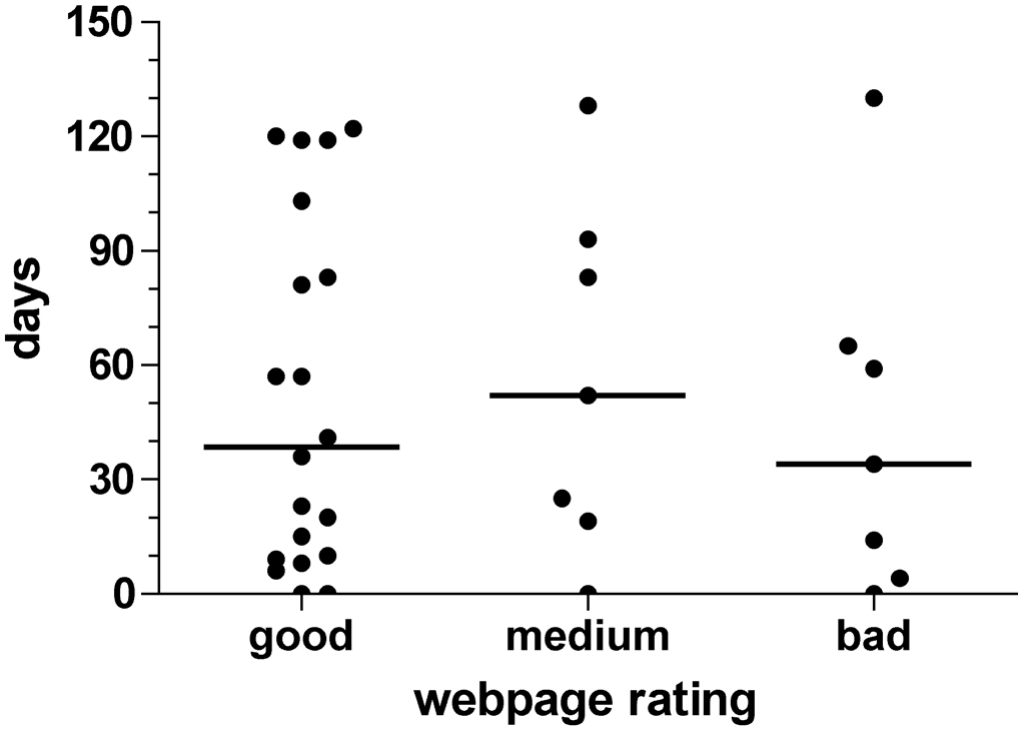
Time-to-final response [days] of N = 35 IRBs as function of quality of available web information about the submission process (p = NS, non-parametric ANOVA).

We evaluated the quality of the web presentations of the various IRBs with regard to their usability and helpfulness to provide transparency towards required information for a successful IRB submission. All IRBs had an internet presentation. Twenty-one/35 (60%) IRB web presentations were considered good, 7/35 (20%) medium, and 7/35 (20%) bad. Of interest, there was no difference in time-to-final response between the three groups (non-parametric ANOVA, Fig 3).

## Discussion

We observed a substantial variation in both work-flow and response time between the IRBs to whom these two studies involving vulnerable subjects were sequentially submitted to after a primary positive vote at the coordinating centers’ IRBs. The median time-to-final decision for both protocols was 38.5 (range 0 to 139) days. Variation in response IRB time is not uncommon. McWilliams et al. carried out a multicenter genetic epidemiology study in cystic fibrosis involving children and adults and reported their experience with 31 IRBs in the United States. Twenty-four IRBs conducted a full review and 7 IRBs considered this study to be of minimal risk and permitted an expedited review process. The mean time for IRB approval in an expedited review was 32.2 days (range 9–72 day) and for a full review 81.9 days (range 13–252 days) [11]. Stair and colleagues published a median time-to-IRB approval of 38 days (IQR 26–62 days) for an interventional multicenter clinical study involving children and adults with asthma at 44 centers in the U.S. [12]. Mansbach reported a median time from initial submission to final approval of 42 days (IQR, 27–61 d) for a prospective, observational study in children presenting with bronchiolitis to emergency departments conducted at 34 centers across the United States [13]. Of interest, studies in vulnerable populations like the two present protocols do not necessarily require a longer time to approval as illustrated by the data from a study by Green at al. Here, the researchers conducted a multicenter observational study related to guideline adherence in diabetes mellitus type 2 and hypertension in 43 Department of Veterans Affairs medical centers in the U.S. Median time to approval for this protocol in common diseases at the various IRBs was 248 days [14].

The request for changes in the present two protocols was focused on the informed consent document, which shows that IRBs fulfill their mission to protect human subjects. Whereas 43% (N = 15/35) of IRBs requested changes to the informed consent documents (N = 15/35, i.e. 43%), very few only (N = 4) asked for changes to the study protocol. This number is lower than in other studies reported in the literature. In the study by Stair et. al, 91% of IRBs requested changes in the consent form, but only 43% of IRBs requested changes to the interventional asthma research protocol [12]. In contrast, in the observational bronchiolitis study by Mansbach, the same amount of changes (56%) was required for research protocol and consent form changes by 62% of the IRBs [13].

One issue that arose in the submission process was the complexity of format and nature of information required that varied among the different IRBs. As a primary source of transparency and information, the internet presentations of the different IRBs were considered and evaluated. Of interest, most websites were rated as “very good” by the submitting individuals which is reassuring for the transparency of the process. The quality of provided information did not influence the time-to-final response.

### Limitations of the present paper

The assessment of time-to-final response does not take into account submission deadlines for upcoming IRB meetings. The management of these deadlines would have been too complex for a multicenter clinical study.

## Conclusion

Both clinical research protocols involving vulnerable populations were well accepted. IRB workflows and decision times varied substantially. Some IRBs accepted a previous IRB decision without the necessity of another reevaluation process. Requested changes focused on the informed consent documents. The strategy with initial submissions to the IRBs at the coordinating site followed by subsequent multicenter submission with the initial positive vote was helpful to reduce the number of changes. The median time-to-final decision for both protocols was 38.5 (range 0 to 139) days. Knowledge of IRB workflows and decision times is helpful for site selection strategies, although the plethora of forms, standards, and local work-flows render submissions of multicenter research protocols complex. A more standardized approach across jurisdictions is desirable.
